# Mortality pattern of cancer patients followed in Palliative Care: a retrospective analysis

**DOI:** 10.1590/1806-9282.20252219

**Published:** 2026-07-10

**Authors:** Filipe Veiga, Cristina Teixeira Pinto

**Affiliations:** 1 University of Porto, Faculty of Medicine – Porto, Portugal.; 2 Entre Douro e Vouga Local Health Unit – Santa Maria da Feira, Portugal.

**Keywords:** Palliative Care, Palliative medicine, Terminal care, Neoplasms

## Abstract

**OBJECTIVE:**

The aim of this study was to identify the places of death of patients with solid cancer under follow-up by a Palliative Care team and to recognize factors that influence the place of death.

**METHODS:**

Retrospective study of cancer patients whose deaths occurred between January 1, 2022, and January 1, 2024, followed up by a Palliative Care team in a Portuguese public hospital. Demographic and clinical data were reviewed through clinical file consultation and were analyzed by Statistical Package for the Social Sciences^®^ v.29, considering a p<0.05 as statistically significant.

**RESULTS:**

The sample included 413 cancer patients; 60.5% were male, and 72.9% were Eastern Cooperative Oncology Group 3–4. The median age was 75 years, and the median follow-up duration in Palliative Care was 40 days. Death in a hospital setting occurred in 64.4% of the patients. The most common types of cancer were gastrointestinal/hepato-bilio-pancreatic (41.6%) and lung cancers (18.6%). Death due to cancer progression was the main cause of death (88.4%). In a multivariate logistic regression, factors associated with higher odds of hospital death were age <75 years (OR 1.88, 95%CI 1.22–2.90) and follow-up duration in Palliative Care <30 days (OR 2.59, 95%CI 1.66–4.04). Death due to cancer progression was associated with lower odds of hospital death (OR 0.03, 95%CI 0.004–0.21).

**CONCLUSION:**

Death in a hospital setting was predominant in this sample, which is in line with outcomes from international studies. These findings highlight the need for early palliative care referral and advance care planning to support patients’ preferences for place of death.

## INTRODUCTION

Available data on the preferences of patients regarding places of death suggest that most cancer patients prefer to die at home^1–5^, as this setting offers more autonomy, control, dignity of care, and less exposure to stressful environments^6,7^. Dying at home, compared to dying in a hospital setting, improves well-being and is more cost-effective, particularly when home-based end-of-life support is provided, as it reduces hospital admissions and unnecessary medical interventions^8,9^. In Portugal, a 2013 study found that 60% of the population would prefer to die at home, yet only 25% of deaths occur there, highlighting a significant gap between preferences and reality^1^. For these reasons, meeting patients’ wishes concerning the place of death is considered an indicator of quality of care^4^.

Aging populations, chronic disease burden, limited home care options, and frequent hospitalizations contribute to higher rates of death of cancer patients in hospital settings^1^.

Palliative Care improves the quality of life of cancer patients through symptom control, communication with patients and caregivers, support for family and/or caregivers, and multidisciplinary work^10^. Early referral to Palliative Care increases the likelihood of dying at home, while longer follow-up with Palliative Care reduces urgent hospital care^11,12^. Conversely, more hospital admissions in the last year of life lower the likelihood of dying at home^11^.

Proximity and availability of healthcare professionals, family support (including being married), higher educational attainment, and living in rural areas are associated with a higher probability of dying at home^11–14^. Among cancer patients, those with solid tumors (particularly gastrointestinal tumors) seem to have a higher probability of dying at home^11–14^.

Data on cancer patients’ places of death are limited and heterogeneous. This study aimed to identify the places of death of patients with solid cancer under Palliative Care at a Portuguese public hospital and to explore factors influencing these outcomes, proposing measures to improve end-of-life support.

## METHODS

A retrospective study was conducted, and data from clinical files of cancer patients followed by a Palliative Care team at a Portuguese public hospital were reviewed between August and December 2023. The study was designed and reported according to the Strengthening the Reporting of Observational Studies in Epidemiology guidelines^15^.

The following inclusion criteria were applied: diagnosis of solid cancer (as an active problem); at least one clinical assessment by the Palliative Care team; referral to Palliative Care between January 1 and December 31, 2022; and occurrence of death between January 1, 2022, and January 1, 2024.

From an initial sample of 645 patients, 65 were excluded for not aligning with the study's asserted time range, 132 for not having a diagnosis of solid cancer, and 35 for dying before the first Palliative Care evaluation. Therefore, the final sample included 413 patients.

Follow-up time with the Palliative Care team included the period from the first contact between the patient and the team until death. The first contact may have occurred in an outpatient setting or at the time of hospitalization.

All data were collected from patients’ medical records, including the following: gender, age, type of malignant tumor, presence of distant metastasis, comorbidities, functional status [measured through the Eastern Cooperative Oncology Group Performance Status (ECOG PS) score^16^], duration of Palliative Care follow-up, type of antineoplastic treatment received in the month preceding death, and date and place of death. ECOG PS was recorded based on the last available assessment within 30 days before death.

The sample was stratified according to the place of death: hospital setting versus outside the hospital setting. To simplify the statistical analysis, patients who died in hospice or long-term care facilities were included in the subgroup of patients who died outside the hospital setting.

Data were analyzed by SPSS^®^ v.29, considering a p-value <0.05 as statistically significant. Statistical methods in the characterization of the sample included frequency measures (absolute and relative frequencies) and association measures (median).

Subgroup analyses were performed to identify demographic and clinical factors that could influence the place of death. In this context, univariate analyses were performed using nonparametric statistical tests (chi-square and Mann-Whitney U). Multivariate logistic regression was also used to identify independent predictors of the place of death, adjusting for potential confounding variables.

This research complied with all ethical guidelines for human experimentation in the Helsinki Declaration and was evaluated and authorized by the Health Ethics Committee of Unidade Local de Saúde Entre o Douro e Vouga (registration n° 48_2023)^17^.

## RESULTS

Among 413 patients, 250 (60.5%) were male. The median age in the last month of life was 75 years (range: 40–100 years), and the proportion of patients <75 years old was 49.9% (n=206).

Gastrointestinal and hepato-bilio-pancreatic cancers were the most common types (n=172, 41.6%), followed by lung cancer (n=77, 18.6%) and genitourinary and gynecologic cancers (n=75, 18.2%). Metastatic tumors were present in 324 patients (78.5%).

Comorbidities were present in 363 patients (87.9%). The most common were cardiovascular risk factors (n=337, 81.6%), including arterial hypertension (n=226, 54.7%) and type 2 iabetes (n=91, 22.0%), heart failure (n=71, 17.2%), venous thromboembolism (n=47, 11.4%), and chronic lung disease (n=46, 11.1%). The presence of a second solid tumor was observed in 20 patients (4.8%), and the most frequent were genitourinary cancers (n=8, 1.9%). Concomitant with the main solid tumor, hematologic tumors were present in 6 patients (1.5%). The presence of terminal comorbidities besides solid cancer was observed in 34 patients (9.0%). The most common were chronic lung disease (n=7, 1.7%), chronic liver disease (n=7, 1.7%), heart failure (n=5, 1.2%), cerebrovascular disease (n=5, 1.2%), and second solid tumor (n=5, 1.2%).

Regarding functional status in the last month, 301 patients had ECOG PS 3–4 (72.9%).

The duration of follow-up in Palliative Care did not follow a normal distribution. The median duration was 40 days (range: 0–625 days), and five patients (1.2%) died on the day of the first contact with the Palliative Care team. The number of patients with follow-up time ≤30 days was 182 (44.1%). Median follow-up duration among patients <75 years was 33.5 days (range: 0–462), and among patients ≥75 years was 50 days (range: 0–625), p<0.002.

With respect to the approach of the oncologic disease in the last month of life, no patients received treatments with curative intent. Seventy-four patients (17.9%) received treatments with palliative intent, chemotherapy being the most frequent (n=28, 6.8%). Invasive procedures were performed in 20 cases (4.8%): 8 (1.9%) were biliary prosthesis placements, and 3 (0.7%) were endoscopic esophageal prosthesis placements. Surgical procedures were performed in four patients (1.0%). In all cases, invasive procedures had palliative intent.

Death in a hospital setting occurred for 266 patients (64.4%), of which 34 deaths (8.2%) were in an emergency department (the remaining patients died in hospital wards, with none in an intensive care unit). Death at home occurred for 121 patients (29.3%), including 8 deaths (1.9%) in nursing homes. A residual number of deaths occurred outside patients’ homes or hospital settings, namely, 12 deaths (2.9%) in long-term care facilities and 14 deaths (3.4%) in hospices.

The most common cause of death was cancer progression (n=365, 88.4%). The remaining deaths (n=48, 11.6%) were mainly due to infectious events (n=34, 8.2% of all deaths, 70.8% of non-cancer deaths).

Table 1 summarizes the demographic and clinical characteristics of the sample.

**Table 1:** Demographic and clinical characteristics of the sample (n=413).

Variables		n (%)
Gender, n (%)		
	Male	250 (60.5)
	Female	163 (39.5)
	Median age, years (range)	75 (40–100)
Tumor type, n (%)		
	GI and HBP	172 (41.6)
	Lung	77 (18.6)
	Gynecologic and GU	75 (18.2)
	Head and neck	21 (5.1)
	Others	68 (16.5)
	Metastization, n (%)	324 (78.5)
ECOG PS, n (%)		
	0–2	112 (27.1)
	3–4	301 (72.9)
Follow-up time in Palliative Care		
	Median, days (range)	40 (0–625)
	≤30 days, n (%)	182 (44.1)
	>30 days, n (%)	231 (55.9)
Place of death, n (%)		
	Hospital setting	266 (64.4)
	Home	121 (29.3)
	Long-term care unit	12 (2.9)
	Palliative Care unit	14 (3.4)
Cause of death, n (%)		
	Progression	365 (88.4)
	Other causes	48 (11.7)

ECOG PS: Eastern Cooperative Oncology Group Performance Status; GI: Gastrointestinal; HBP: hepato-bilio-pancreatic; GU: genitourinary.

The median age of the patients who died in a hospital (72 years, range: 40–100) was statistically lower than the median age of patients who died outside a hospital setting (78 years, range: 41–96), p=0.001. Median follow-up time by the Palliative Care team was also lower among patients who died in a hospital (28 days, range: 0–625, vs. 63 days, range: 1–482, p<0.001).

Univariate analysis identified the following factors as associated with higher odds of hospital death: age <75 years (p=0.001), ECOG PS 0–2 (p=0.028), follow-up time in Palliative Care ≤30 days (p<0.001), absence of comorbidities (p=0.045), absence of arterial hypertension (p=0.019), absence of type 2 diabetes (p=0.007), and absence of pressure ulcers (p=0.040). Death due to cancer progression was associated with higher odds of dying outside the hospital setting (p<0.001).

In the multivariate logistic regression, variables associated with higher odds of dying in a hospital in this sample were a short time of follow-up (<30 days) by the Palliative Care team (OR 2.586, 95%CI 1.656–4.036, p<0.001) and age <75 years (OR 1.881, 95%CI 1.219–2.904, p=0.004) (Table 2). Death due to cancer progression was associated with higher odds of dying outside the hospital setting (OR 0.029, 95%CI 0.004–0.214, p<0.001) (Table 2).

**Table 2:** Multivariate logistic regression analysis of factors associated with death in a hospital setting.

Variables associated with death in a hospital setting		Odds ratio	95%CI	p-value
Factors associated with a positive effect				
	Follow-up time in Palliative Care <30 days	2.586	1.656–4.036	<0.001
	Age <75 years	1.881	1.219–2.904	0.004
A factor associated with a negative effect				
	Death due to cancer progression	0.029	0.004–0.214	<0.001

CI: confidence interval.

## DISCUSSION

Evidence shows that the place of death may influence the quality of life of cancer patients^18^. In this study most patients died in a hospital setting, and home was the most frequent location among those who died outside a hospital setting. These findings align with previous data and raise concern, as evidence highlights a low correlation between preferred and actual place of death, indicating that patients’ end-of-life preferences are often unmet.

Age <75 years was associated with increased odds of dying in a hospital setting. The impact of age on the place of death is not consistent according to the available evidence^11–13^. In our study, the higher proportion of deaths in a hospital setting among patients <75 years may be justified by the fact that this group had a significantly lower median follow-up duration in Palliative Care compared to the group of patients ≥75 years. Another reason for this finding is that younger patients may have shorter palliative care follow-up due to later referral, possibly because they are more likely to receive disease-directed therapies until late in the disease course^11–13^.

Shorter follow-up time in Palliative Care (<30 days) increased the odds of dying in a hospital setting. This association reinforces the need for early referral to a Palliative Care team, thus speeding the approach to palliative cancer patients and their caregivers^2,12,13^.

The finding that the absence of comorbidities was associated with hospital death in univariate analysis was unexpected and may reflect residual confounding or chance, as it did not remain significant in multivariate analysis.

The only variable associated with lower odds of dying in a hospital was death due to cancer progression, consistent with evidence that the absence of acute complications reduces hospital admissions^19^. This suggests that preventing and managing acute events in the community may reduce hospital deaths and support patients’ preferences^2,19^.

Based on the outcomes of this study, some measures can be taken to improve Palliative Care and honor cancer patients’ preferences at the end of life. The following items could be the main practical implications: prioritizing personalized advance care plans; planning the provision of Palliative Care; identifying the preferences and wishes of the cancer patient; increasing the knowledge of healthcare professionals about Palliative Care; and guaranteeing global coverage of community-based Palliative Care to support palliative cancer patients who wish to die at home^2,20–22^.

This study has a retrospective and a single-center design. Those factors limit generalizability to other regions or countries with different palliative care models. A larger sample in the context of a multicenter study is needed to strengthen the findings of our study and to better study potential determinants of the place of death, namely, the type of cancer, the metastasis pattern, and the symptom burden of the disease (because it can influence morbidity and prognosis).

Some potential determinants of the place of death were not analyzed in this study. Those determinants include data on family and/or caregiver support, socioeconomic and educational status, housing conditions, and the level of symptom control.

Another important limitation of this study is the fact that data were collected from patients’ medical records. Therefore, the absence of some information due to omission or recording errors prevented obtaining data from other variables that could be important for the purpose of the study. The absence of information regarding the patients’ preferred place of death and the existence or absence of advance directives is the main limitation in this context.

## CONCLUSION

Most cancer patients in this study died in a hospital setting, despite evidence that home death is often preferred. Shorter Palliative Care follow-up and younger age were associated with hospital death, while death due to cancer progression was associated with home death. These findings highlight the need for early Palliative Care referral, advance care planning, and community-based support to align end-of-life care with patients’ preferences. Further research should explore barriers to home death and the impact of family support and socioeconomic factors.

## Data Availability

The datasets generated and/or analyzed during the current study are available from the corresponding author upon reasonable request.
